# Value of Histopathologic Examination of Uterine Products after First-Trimester Miscarriage

**DOI:** 10.1155/2014/863482

**Published:** 2014-06-26

**Authors:** Sharifa Ali Alsibiani

**Affiliations:** Department of Obstetrics and Gynaecology, Faculty of Medicine, King Abdulaziz University, P.O. Box 122413, Jeddah 21332, Saudi Arabia

## Abstract

The main rationale of routine histopathologic examination of products after first-trimester miscarriages is to detect an ectopic pregnancy or a molar pregnancy, which require further management. An alternative approach is to examine the products only when there is a definite indication. As there is no agreement, we aimed to study whether routine histopathological examination of tissues obtained after first-trimester miscarriage is of any clinical value in our populations. Medical records of all (558) patients with a diagnosis of first-trimester miscarriage over 4 years (2007–2010) at King Abdulaziz University Hospital, Jeddah, Saudi Arabia, were reviewed. Histopathologic examination confirmed products of conception in 537 (96.2%) patients, no products of conception in 17 (3%) patients, molar pregnancy in 2 (0.4%) patients, and decidual tissues without chorionic villi (Arias-Stella reaction) in 2 (0.4%) patients. After clinical correlation, only one unsuspected partial molar pregnancy was diagnosed by histopathology examination. Conclusion is that it does not appear reasonable to perform histopathological examination routinely after all first-trimester miscarriages in our studied population. We recommend that histopathological examination be performed in select instances: when the diagnosis is uncertain, when fewer tissues have been obtained during surgery, when unexpected pathology was seen, when ultrasound suggests a molar pregnancy, or when patients are considered at high risk for trophoblastic disease.

## 1. Introduction

The United States Centers for Disease Control and Prevention and the World Health Organization define miscarriage as pregnancy termination before 20-week gestation or a fetus born weighing less than 500 g [1]. Miscarriage is one of the most common first-trimester conditions encountered by obstetricians and gynecologists. Approximately 10–20% of clinical pregnancies are lost spontaneously during the first trimester [2], which is defined as the first 12–14 weeks of gestation [3]. Traditionally, most women who had spontaneous miscarriage have undergone surgical uterine evacuation of retained products of conception (RPOC). In recent years, more women are being treated on an outpatient basis and more refined diagnostic techniques and therapeutic interventions are being applied [3].

In most centers, it is a routine practice to submit tissues obtained by uterine evacuation for histopathologic examination to confirm the presence of intrauterine fetal tissue. However, there is little agreement about the value of this practice. The main rationale is to detect an ectopic pregnancy, which requires immediate further management, or a molar pregnancy, which necessitates special followup. Other reasons include detecting surgical complications, such as incomplete or failed pregnancy evacuation; determining the cause of recurrent pregnancy loss; or detecting unexpected fetal pathology [4]. Routine histopathologic examination of uterine products passed spontaneously or evacuated surgically or medically is beneficial in protecting obstetrician and gynecologist from medico legal recrimination, but it is unclear whether this practice is medically justified. An alternative approach is to examine the products only when there is a definite indication, such as when there is uncertainty about the diagnosis, either preoperatively or at the time of surgery [4].

At our institution, the RPOC passed spontaneously or removed during surgical or medical evacuation are routinely subjected to histopathological examination. The aim of this study is to determine whether histopathological examination of tissues obtained from first-trimester miscarriage is of any clinical value.

## 2. Methods

In this retrospective study, we examined the records of 558 patients admitted to the King Abdulaziz University Hospital, Jeddah, Saudi Arabia, from January 1, 2007, to December 31, 2010, with the diagnosis of first-trimester (5–14 weeks) spontaneous miscarriage. A total of 620 histopathological reports were reviewed, including two to three specimens for 44 (7.8%) patients. Clinical data, including age, parity, gestational age, clinical diagnosis, method of evacuation, and other relevant information, were collected.

At King Abdulaziz University Hospital, all tissues obtained from the time of the end of pregnancy were placed in a solution of 10% formaldehyde and sent to the histopathology laboratory. The samples were examined macroscopically by a histopathologist before being embedded in paraffin blocks for further processing. The paraffin blocks were cut by a microtome into 4 mm sections, which were subsequently stained with haematoxylin and eosin. The sections were examined microscopically by a histopathologist, who was usually the same individual who performed the macroscopic examination. Averages of five blocks were examined for each patient, and additional blocks were sometimes required to detect chorionic villi. An intrauterine pregnancy was confirmed if fetal tissues, trophoblasts, or chorionic villi were identified in addition to other tissues, such as deciduae or secretory endometrium [4]. A recent intrauterine pregnancy was suggested by the presence of deciduae or the identification of an Arias-Stella reaction, but this did not exclude the possibility of an ectopic pregnancy. For each patient, the report included a note about the absence or presence of trophoblastic disease, including a molar pregnancy. Trophoblastic disease was classified according to Rosai [5] and the absence or presence of a malignant trophoblastic neoplasm was noted.

## 3. Results

During the study period, a total of 558 women were admitted with the diagnosis of first-trimester miscarriage. Their mean age was 33.7 ± 7.5 years (±standard deviation (SD); range: 14–48 years) and mean parity was 3.1 ± 2.2 (±SD; range: 0–11). A history of miscarriage was present in 0.45 ± 1.0 patients (mean ± SD; range: 0–7).

As shown in Table 1, the clinical diagnosis on admission was incomplete miscarriage in 253 (45.3%) patients, missed miscarriage in 253 (45.3%) patients, blighted ovum in 28 (5.0%) patients, query complete miscarriage in 15 (2.7%) patients, inevitable miscarriage in 6 (1.1%) patients, and septic pregnancy in 3 (0.5%) patients. Complete miscarriage was confirmed in 62 patients (11.1%) based on clinical and ultrasound findings; these women underwent no intervention. Uterine evacuation was performed in 496 (88.9%) patients. In all except one, the method of evacuation was surgical, either as a sole procedure or by misoprostol 800 *μ*g vaginally. The remaining patient underwent only medical evacuation of products of conception (Table 2).

A total of 620 specimens were examined histopathologically, including two to three specimens for 44 (7.8%) patients. For the latter patients, the first specimen was the tissue obtained when patients spontaneously miscarried at home or on presentation to the hospital, and the second specimen was obtained from the same patient during surgical evacuation or when tissue was spontaneously passed after hospital admission. When more than one specimen was obtained from the same patient, the final histopathologic diagnosis was used for data analysis.

The histopathologic examination results were as follows: RPOC, 537 (96.2%) patients; no RPOC, 17 (3%) patients; complete molar pregnancy, 1 (0.2%) patient; partial molar pregnancy, 1 (0.2%) patient; and decidual tissue without chorionic villi (Arias-Stella reaction), 2 (0.4%) patients (Table 3).

The two patients whose examination exhibited an Arias-Stella reaction were determined to have missed miscarriage, as both had previous ultrasounds that confirmed intrauterine pregnancies. Both had a history of passing tissue and were diagnosed clinically with incomplete miscarriage. One brought the passed tissue with her to the hospital, which showed RPOC on histopathologic examination. Both underwent surgical uterine evacuation because of retained products of conception detected by admission ultrasound; it was these products that showed the Arias-Stella reaction.

The one complete molar pregnancy detected by histopathologic examination was also diagnosed preoperatively by ultrasound and high beta-hCG levels. This patient was managed according to the recommended protocol. The woman with a partial molar pregnancy was the only patient with unexpected pathology not diagnosed by ultrasound. She was initially diagnosed with a missed miscarriage by ultrasound and then miscarried spontaneously. The passed tissue was sent for histopathologic examination, which was later reported as showing query partial molar pregnancy. After further followup, ultrasound revealed retained products, and the patient was diagnosed with an incomplete miscarriage and underwent medical evacuation with misoprostol. Histopathologic examination of the tissue passed this time (second specimen) showed no RPOC, and posttermination ultrasound confirmed an empty uterine cavity. No specific followup was done. After almost one year, she presents to outpatient pregnant with healthy fetus of 16 weeks of gestation.

## 4. Discussion

Miscarriage is a common condition, and like many disorders, the correct diagnosis is essential for proper management. Of particular concern is the possibility that it may be erroneously diagnosed when another condition that requires immediate treatment or long-term followup exists, such as an ectopic pregnancy or gestational trophoblastic disease. Ultrasound, quantitative beta-human chorionic gonadotropin (beta-hCG) testing, and histopathologic examination of the uterine products are diagnostic tools to determine the type of miscarriage and to differentiate miscarriages from other conditions.

Histopathologic examination of the tissue passed by a spontaneous miscarriage or removed during uterine evacuation is common, aimed at detecting unsuspected conditions (e.g., molar or ectopic pregnancy) and diagnosing surgical complications (e.g., incomplete or failed pregnancy evacuation). The practice of routine histopathologic evaluation of tissue obtained at the time of miscarriage has been the subject of debate. Some authorities [6–10] suggest that a sample of tissue from all uterine evacuations (either spontaneous or surgical/medical) be submitted for histological examination to exclude an ectopic pregnancy or gestational trophoblastic diseases, to investigate whether the miscarriage was due to dysmorphic or disruptive causes, and sometimes to simply confirm that pregnancy had occurred.

Previous researches have sought to determine the utility and cost-effectiveness of histopathologic evaluation of first-trimester miscarriage tissue. Some studies [7–9] have found numerous pathologies in tissue from first-trimester miscarriage, including molar pregnancy, ectopic pregnancies, partial hydatidiform mole, complete hydatidiform mole, exaggerated placentalsite, placental site trophoblastic nodule, and decidual tissue. The authors of these studies concluded that routine histopathologic assessment of products of first-trimester spontaneous miscarriage canlead to the diagnosis of important pathologies, more effectively identify ectopic pregnancies or infections than clinical or other laboratory criteria, and may reduce mortality. By contrast, others have recommended against routine histopathological examination of tissues obtained from first-trimester miscarriage.

Heath et al. [4] found only two (0.13%) molar changes and two (0.13%) tubal ectopic pregnancies among 1576 patients on histopathologic examination of the tissue removed during therapeutic miscarriage or emergency uterine evacuation for a spontaneous miscarriage. These authors recommended histopathology only for patients with an uncertain preoperative diagnosis or those in whom a small amount of tissue was collected or trophoblastic tissue was identified during evacuation.

Histologic examination is a reliable method of diagnosing pathologic pregnancies [6]. Excluding hydatidiform mole histologically has obvious medical value, as it rules out the possibility of persistent trophoblastic disease or choriocarcinoma [6]. To rule out gestational trophoblastic disease after miscarriage, the pathologist should examine all recovered material. The material should be examined macroscopically and microscopically with at least five cassettes if the appearance suggests gestational trophoblastic disease [6]. Histologic examination of 670 unselected cases at Charing Cross Hospital of London showed that 120 (18%) patients with unsuspected molar pregnancy were diagnosed with moles based on abundant trophoblast in early pregnancy or the presence of hydrops [6].

A hydatidiform mole is usually first suspected by ultrasound and later confirmed by histopathology. A retrospective analysis of 90 cases of sonographically suspected and histologically proven hydatidiform mole showed that ultrasonography was more reliable for diagnosing complete molar than partial molar pregnancy [11]. The sensitivity of ultrasound for predicting hydatidiform mole overall was 44%–95% for complete hydatidiform and only 20% for partial hydatidiform [11]. Similarly, Sebire et al. [12] noted that molar pregnancy was suspected on ultrasound in only 34% of 155 women with molar pregnancies confirmed by histopathology.

Although the universal practice of histologically examining any tissue passed or removed during a spontaneous or therapeutic miscarriage should prevent first-trimester molar pregnancies from being undetected, the significance of a partial mole is disputed [4] and it remains unclear whether women actually benefit from the results of these histological reports.

The incidence of gestational trophoblastic disease in the region is a factor that may influence the value of routine histopathologic examination of obtained tissues. In Asia, the incidence of hydatidiform mole is as high as 1 in 80 pregnancies, whereas in the western world, it is 1 in 500–1500 pregnancies [13, 14]. The incidence in Saudi Arabia is similar to the latter: 1 in 452–1098 pregnancies, and it has decreased over time, paralleling sociomedical improvements [15–17].

In our study, RPOC were undetected in 17 patients, all of whom were diagnosed by ultrasound with various forms of retained products. Part of this discrepancy may relate to the ultrasound criteria used to define “retained products.” The criteria have varied in previous studies [16]. One study included patients with an anterior-posterior (AP) tissue diameter of 15–50 mm with ultrasound review at 3 days (efficacy 71%) [10]. When ultrasound assessment of the uterine cavity shows heterogeneous shadows with a maximum AP diameter of 15 mm or less, genuine retained products are less likely to be confirmed histologically [10]. Our 17 patients were confirmed to have had a complete miscarriage, as ultrasound misinterpreted the usual blood clots for retained products. The Turkish study [8] discussed above reported a similar finding in 128 (8.0%) patients, which was attributed to the cut-off value of 15 mm AP diameter being not optimal and/or patients with an incomplete abortion fully aborting by the time of their surgical evacuation.

Our observation that more than one tissue sample was examined for 44 patients (7.9%) deserves discussion. Such additional examination(s) increase costs and laboratory personnel's time and may lead to misdiagnosis. Pregnant women should be advised that if they pass tissues at home, they should bring these with them to the hospital. If or when histopathologic examination is performed, all tissues passed or collected during miscarriage for a given patient should be sent to the laboratory in the same container. All of these materials should be examined both macroscopically and microscopically. Patient care should be based on the histological findings, but before that the assessment should be based on the overall clinical picture.

The cost of a pathohistological examination at our institution is 500 Saudi Riyals ($133 US), so the total cost for 620 specimens was 310,000 Saudi Riyals ($82,666.00 US). Because of the cost, unclear usefulness of diagnosing a partial molar pregnancy, and also in view of the trophoblastic diseases are uncommon in Saudi Arabia, even more the last local studies showed decreasing in their incidence over time in Saudi Arabia, it does not appear reasonable for all women with first-trimester miscarriage at our institution to undergo tissue histopathological examination to identify only one unsuspected pathology (a partial mole) in 620 specimens.

*In conclusion*, considering the findings of our study, the cost of histopathological examinations, and the low incidence of molar pregnancies in Saudi Arabia, we can say it may not appear reasonable to perform these examinations routinely after all first-trimester miscarriage. We recommend that histopathological examination be performed in select instances: when the diagnosis is uncertain preoperatively, when fewer tissues than expected have been obtained, when ultrasound suggests a molar pregnancy, when patients are considered of high risk for trophoblastic disease, or when inspection during surgery suggests unexpected pathology.

To our knowledge, this study is the first local study that evaluated routine histopathological examination after first-trimester miscarriage. It is a hospital-based study and its findings increase the importance of a nationwide study to guide the revision of practice at a national scale in Saudi Arabia.

## Figures and Tables

**Table 1:** Type of abortion on admission.

Type of abortion	Number (percentage)
Incomplete	253 (45.3%)
Missed	253 (45.3%)
Blighted ovum	28 (5.0%)
Complete	15 (2.7%)
Inevitable	6 (1.1%)
Septic	3 (0.5%)

Total	558 (100%)

**Table 2:** Method of pregnancy termination.

Method of termination	Number (percentage)
Spontaneous (no intervention)	62 (11.1%)
Surgical	495 (88.7%)
Medical	1 (0.2%)

Total	558 (100%)

**Table 3:** Histopathology report diagnosis.

Histopathological diagnosis	Number (percentage)
POC∗	537 (96.2%)
No POC	17 (3.0%)
Arias-Stella reaction	2 (0.4%)
Partial mole	1 (0.2%)
Complete mole	1 (0.2%)

Total	558 (100%)

*Products of conception.
